# Tissue Doppler Imaging as a predictor of therapy-related cardiac dysfunction in breast cancer patients

**DOI:** 10.1007/s10554-025-03439-1

**Published:** 2025-06-23

**Authors:** Beatriz Piñeiro-Lamas, Ana López-Cheda, Ricardo Cao, Rocío Lesta-Mellid, Alberto Bouzas-Mosquera, Cayetana Barbeito-Caamaño

**Affiliations:** 1 Grupo de Modelización, Optimización e Inferencia Estatística, Departamento de Matemáticas, Facultade de Informática, Universidade da Coruña, CITIC, Campus de Elviña, 15071 A Coruña, Spain; 2 Servicio de Oncología, Complexo Hospitalario Universitario de A Coruña, 15006 A Coruña, Spain; 3 Servicio de Cardiología, Unidad de Imagen y Función Cardíaca, Complexo Hospitalario Universitario de A Coruña, Instituto de Investigación Biomédica de A Coruña (INIBIC), 15006 A Coruña, Spain

**Keywords:** Cardiotoxicity, Cure models, Echocardiography, Survival analysis, Tissue Doppler Imaging

## Abstract

Although early detection and prediction of cancer therapy-related cardiac dysfunction (CTRCD) have been widely studied in the literature in breast cancer patients, little is known about the potential predictive role of Tissue Doppler Imaging (TDI) as a risk factor. The purpose of this study is to assess the predictive role of Pulsed-waved TDI in predicting cardiotoxicity in breast cancer patients. In total, 270 breast cancer patients, 27 of whom experienced CTRCD during the follow-up period, were enrolled. Echocardiography was performed at baseline (before the beginning of the treatment), and TDI images were obtained. Other clinical variables were also measured. Significant differences were found between the TDI images of patients who developed cardiotoxicity and those of patients who did not, particularly around the $\:{E}^{{\prime\:}}$ wave. Specifically, patients with CTRCD had a smoother and later $\:{E}^{{\prime\:}}$ wave. The variable $\:{v}_{{E}^{{\prime\:}}}$, which accounts for both the peak velocity of the $\:{E}^{{\prime\:}}$ wave and the timing within the cardiac cycle when this peak is reached, was found to be highly informative. Patients with a $\:{v}_{{E}^{{\prime\:}}}$​ value above the threshold of 0.052 have a higher risk of CTRCD, which supports its potential as an early indicator of the risk of cardiotoxicity. The $\:{v}_{{E}^{{\prime\:}}}$ in the baseline TDI is significantly associated with the CTRCD. Specific characteristics of the ${E}^{\prime}$ wave, such as the moment in the cycle when its peak occurs and its velocity, may be used in clinical practice to predict cardiovascular side effects in patients undergoing breast cancer treatment.

## Introduction

Breast cancer is the most common cancer type among women. According to the World Health Organization, 2.3 million women worldwide were diagnosed with breast cancer in 2022, and 670,000 died from the disease [1]. Although breast cancer survival rates have improved over the past few decades due to advances in the field of oncology, some patients develop cardiovascular side effects during or after treatment. Anthracyclines (AC) and HER2-targeted therapies are commonly used as treatments for breast cancer and are well known to increase the risk of cardiovascular events [2, 3]. Cardiovascular toxicity can occur in many ways, but one of the most notable manifestations is cardiac systolic dysfunction. The development of cancer therapy-related cardiac dysfunction (CTRCD) has important prognostic implications, and CTRCD may progress to heart failure over time, making early detection an ongoing challenge. Baseline echocardiography and clinical evaluation are mandatory to predict the risk of CTRCD. Previous studies have identified clinical factors such as age, hypertension or previous anthracycline treatment, among others, as risk factors [4]. However, these factors may not be sufficient to predict which patients will develop CTRCD during treatment, and laboratory and imaging factors must be considered. The left ventricular ejection fraction (LVEF) has traditionally been the most widely studied imaging parameter, and a low or low-normal baseline value is known to identify patients at highest risk of CTRCD [5]. Global longitudinal strain (GLS) has emerged as an early and sensitive predictor of cardiotoxicity, often detecting cardiac dysfunction before changes in LVEF become apparent during follow-up [6, 7]. However, there is currently insufficient evidence on its usefulness in predicting the risk of cardiotoxicity at baseline. In addition, not all hospitals have access to the specialized equipment and software needed for strain analysis. Other echocardiography parameters have been less studied. Pulse-waved Tissue Doppler Imaging (TDI) is routinely performed and measures the velocity of myocardial motion, providing valuable information about the function and mechanics of the heart. This information, which reflects the condition of the heart just before treatment begins, may help in the early identification of CTRCD. The analysis of diastolic function through the TDI in the baseline echocardiogram, performed before starting oncological treatment, could provide valuable information for identifying patients with a higher risk of developing cardiotoxicity during follow-up. However, the use of image covariates as risk factors has not been broadly examined in previous studies.

## Materials and methods

### Study population

**Fig. 1:**
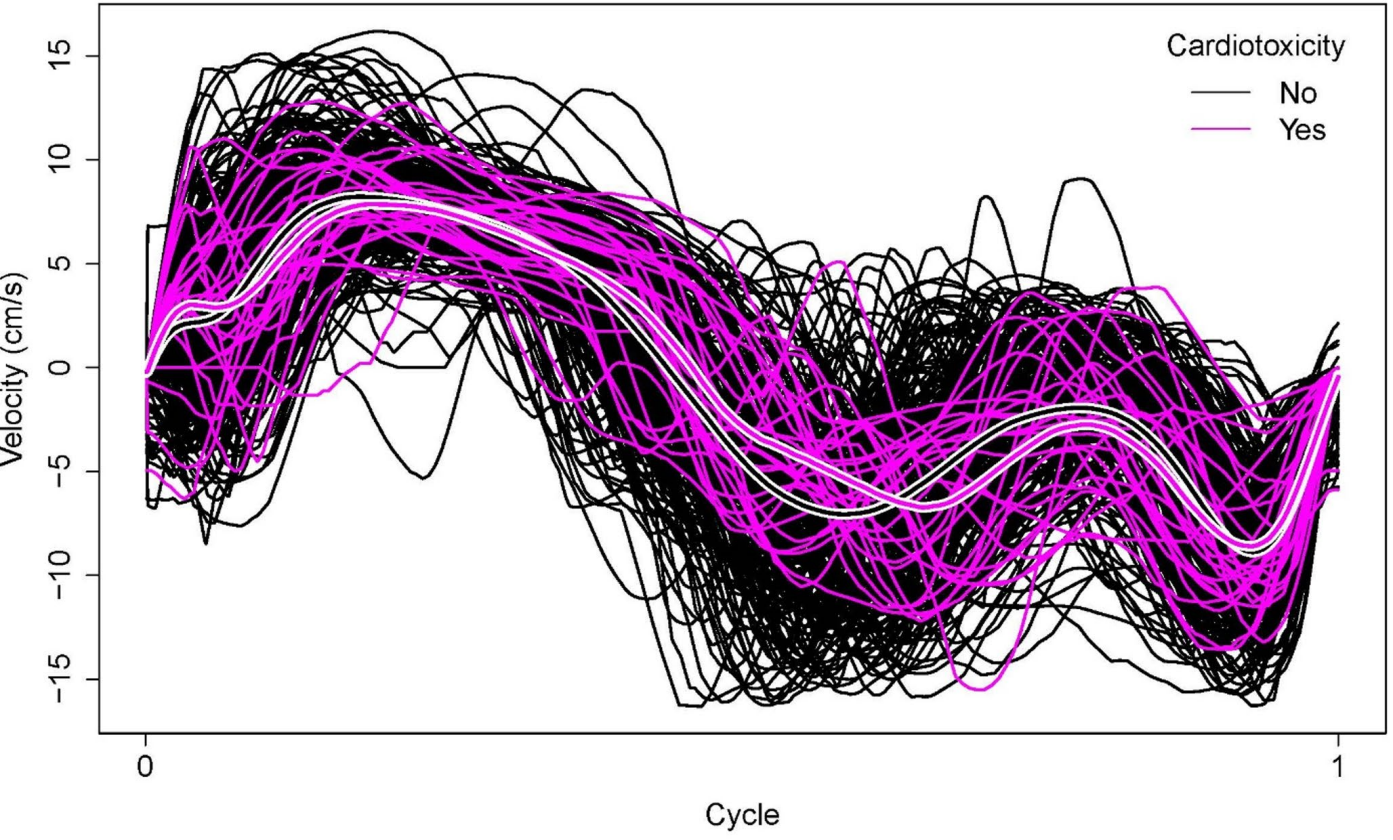
TDI cycles depending on the cardiotoxicity status. The cycles of patients who experienced cardiotoxicity during the follow-up period are shown in magenta, and the cycles of the patients who did not experience this side effect are shown in black. The thick lines with white borders represent the mean cycle of each group. For patients with cardiotoxicity, the $\:{E}^{{\prime\:}}$ wave seems to be smoother and slightly shifted to the right

In total, 270 women who were diagnosed with breast cancer between 2007 and 2021 and who underwent baseline echocardiography at the Imaging Unit of the University Hospital of A Coruña (Spain) before starting treatment with AC and/or HER2-targeted therapies were included. All patients were evaluated with the same echocardiography equipment. Patients with atrial fibrillation and those without at least one follow-up echocardiogram were excluded. For each patient, a complete analysis of diastolic function was performed with a baseline echocardiogram by means of TDI. Cardiotoxicity was defined as a decrease in LVEF to a value below 50% with a reduction of at least 10% points from the baseline LVEF during follow-up, corresponding to moderate-severe toxicity [8]. This dataset is freely available in the online repository [9] and further detailed in the related work [10]. For each woman, the dataset includes binary and numerical baseline covariates, a time variable and an noncensoring indicator. For patients who experienced cardiotoxicity, the variable time contains the time (in days) until its appearance. For patients who did not experience cardiotoxicity, it reflects the length of the follow-up period. The median follow-up time for the entire cohort was 437 days, and the median time to onset was 316 days among patients who developed CTRCD. The noncensoring indicator denotes whether the patient did (27) or did not (243) experience CTRCD during the follow-up period. Additionally, the baseline TDI is available for each patient. An image preprocessing algorithm [10] was used to extract the underlying velocity function from the TDI images, with a focus on a single cardiac cycle period (Fig. 1). The corresponding functions, discretized into 1001 equispaced points in the interval [0,1], can be found in the repository file BC_cardiotox_functional_variable.csv, available in [9].

### Statistical analysis

To assess susceptibility to CTRCD, we define a binary variable, $\:\nu\:$, such that $\:\nu\:=0$ if the subject is susceptible and $\:\nu\:=1$ if not (in this case, the patient is said to be cured for CTRCD). Importantly, $\:\nu\:$, known in statistics as the cure status (understanding by cure the nonsusceptibility to the event), is only partially observed due to censoring. This represents a significant challenge in this study. The noncensored observations (that is, the patients who experienced cardiotoxicity during the follow-up period) are known to be susceptible ($\:\nu\:=0$). However, it is unknown whether a censored individual will eventually be cured ($\:\nu\:$ is missing) since there is the possibility that cardiotoxicity would have occurred if the follow-up period had been longer, although the patient may also be nonsusceptible or cured.

To analyze differences between susceptible and cured patients, we defined two mean TDI functions: $\:{{\upmu\:}}_{0}\left(\text{t}\right)$ for susceptible patients ($\:\nu\:=0$) and $\:{{\upmu\:}}_{1}\left(\text{t}\right)$ for cured patients ($\:\nu\:=1$). We then introduced three discrepancy measures to quantify the discrepancies between these functions:

- $$\:{D}_{1}={\int}_{0}^{1}\left|{{\upmu}}_{0}\left(\text{t}\right)-{{\upmu}}_{1}\left(\text{t}\right)\right|\text{d}\text{t}$$ 
- $$\:{D}_{2}={\int}_{0}^{1}{\left({{\upmu}}_{0}\left(\text{t}\right)-{{\upmu}}_{1}\left(\text{t}\right)\right)}^{2}\text{d}\text{t}$$ 
- $\:{D}_{3}={t}_{{{\upmu}}_{0},{\text{m}\text{i}\text{n}\text{E}}^{{\prime}}}-{t}_{{{\upmu}}_{1},{\text{m}\text{i}\text{n}\text{E}}^{{\prime}}}$, where $\:\:{t}_{{{\upmu}}_{0},{\text{min}\text{E}}^{{\prime}}}$ and $\:\:{t}_{{{\upmu}}_{1},{\text{min}\text{E}}^{{\prime}}}$denote the moment in the cycle (between 0 = start to 1 = end of cycle) when the minimum of the $\:{E}^{{\prime\:}}$ wave of $\:{{\upmu}}_{0}\left(\text{t}\right)$ and $\:{{\upmu\:}}_{1}\left(\text{t}\right)$ are reached, respectively.

While the first two discrepancies consider the entire cycle and account for both vertical and horizontal differences between them, the third one is more specific: it focuses on the timing of the $\:{E}^{{\prime\:}}$ wave peak within the cycle. In practice, once both mean functions are calculated, estimates for $\:{D}_{1},\:{D}_{2}$ and $\:{D}_{3}$ can be obtained. To check whether the discrepancy between the functions of both groups is significant or can be due to randomness, a permutation test for the comparison of the mean functions of two groups is proposed. Let $\:{m}_{1}$ be the dimension of the first group, $\:{m}_{2}$ be the dimension of the second group and $\:{M=m}_{1}+{m}_{2}$. Moreover, let $\:B$ be the number of permutations. For each $\:b=1,\dots\:,\:B$, the permutation test consists of the following steps:

1. From the $\:M$ functions, randomly select two subsets of cardinal $\:{m}_{1}$ and $\:{m}_{2}$ (without replacement).
2. Calculate the sample mean function of each subset, $\:{\widehat{\mu\:}}_{0,b}\left(t\right)$ and $\:{\widehat{\mu\:}}_{1,b}\left(t\right).$
3. Estimate $\:{D}_{1},\:{D}_{2}$ and $\:{D}_{3}$ from both mean functions:

$$\:{\widehat{D}}_{1,b}={\int}_{0}^{1}\left|{\widehat{\mu}}_{0,b}\left(t\right)-{\widehat{\mu}}_{1,b}\left(t\right)\right|\text{d}\text{t}$$

$$\:{\widehat{D}}_{2,b}={\int}_{0}^{1}{\left({\widehat{\mu}}_{0,b}\left(t\right)-{\widehat{\mu}}_{1,b}\left(t\right)\right)}^{2}\text{d}\text{t}$$

$$\:{\widehat{D}}_{3,b}={t}_{{\widehat{\mu\:}}_{0,b},{\text{m}\text{i}\text{n}\text{E}}^{{\prime\:}}\:}-\:{t}_{{\widehat{\mu}}_{1,b},{\text{m}\text{i}\text{n}\text{E}}^{{\prime}}}$$

By repeating the previous steps $\:B$ times, three samples are obtained: $\:{\widehat{D}}_{\text{1,1}},\:\dots\:\:,\:{\widehat{D}}_{1,B}$, $\:{\widehat{D}}_{\text{2,1}},\:\dots\:\:,\:{\widehat{D}}_{2,B}$ and $\:{\widehat{D}}_{\text{3,1}},\:\dots\:\:,\:{\widehat{D}}_{3,B}$. They provide the empirical distribution of the distance between the mean cycles of cured and susceptible women under the null hypothesis that they belong to the same population.

Additionally, we create a receiver operating characteristic (ROC) curve for a new parameter derived from the TDI $\:{E}^{{\prime\:}}$ wave based on the permutation test outcomes. The area under the curve (AUC) quantifies the parameter’s ability to distinguish between patients who developed cardiotoxicity and those who did not. To determine the optimal cutoff point that maximizes sensitivity and specificity, we used Youden’s index.

To assess the associations between baseline characteristics and time to cardiotoxicity, proportional hazards Cox regression models were fitted. The specific variables considered in this analysis are as follows:

- The LVEF was discretized into the categories < 50%, 50 − 54%, and > 54%.
- Age was discretized into the categories < 50, 50 − 64, 65 − 79, and ≥ 80.
- Prior treatment: The variables antiHER2prev, ACprev and treatprev indicate whether the patient received prior treatment with antiHER2, anthracyclines or any of both.
- A new TDI-derived parameter defined in the Results section, which is based on $\:{E}^{{\prime\:}}$ wave characteristics, is discretized into “low” and “high” using the optimal cutoff identified from the ROC curve analysis.

The first three variables were selected with a focus on risk factors identified as “high” or “very high” for cardiotoxicity induced by anthracyclines and antiHER2 therapies in the 2022 ESC Guidelines on cardio-oncology [5]. Continuous variables are categorized according to established reference levels. The results are presented with hazard ratios (HRs), 95% confidence intervals (CIs) and $\:p$-values. The proportional hazards assumption was assessed for each Cox model using Schoenfeld residuals. Statistical analyses were performed using R software (version 4.3.1 [11]), and a $\:p$-value < 0.05 indicated statistical significance.

## Results

### Baseline characteristics

The baseline clinical characteristics and cardiac risk factors for the 270 patients included in this study, stratified by CTRCD development during follow-up, are presented in Table 1. Patients who developed CTRCD (27) were slightly older (59.15 ± 8.86 vs. 57.43 ± 10.96 years) and had a lower baseline LVEF (62.42 ± 6.45% vs. 66.01 ± 7.24%) than did those who did not develop CTRCD (243), although these values were normal in both groups. Prior treatment with anthracyclines was more common in the CTRCD group (29.17% vs. 9.82%), as was prior anti-HER2 therapy (12.5% vs. 1.79%).

**Table 1:** Baseline clinical characteristics and cardiac risk factors in patients with and without CTRCD. Continuous variables are presented as mean ± sd, whereas categorical variables are presented as counts (percentages). Left ventricular ejection fraction is indicated by LVEF. AntiHER2prev indicates that the patient received prior treatment with antiHER2 therapies; acprev indicates that the patient received prior treatment with anthracyclines. The variable $\:{v}_{{E}^{{\prime\:}}}=\:\frac{{t}_{{E}^{{\prime\:}}}}{{|E}^{{\prime\:}}|}$, where $\:{t}_{{E}^{{\prime\:}}}$ is the timing within the cycle (between 0 and 1) when the peak of the $\:{E}^{{\prime\:}}$ wave occurs, and $\:{|E}^{{\prime\:}}|$ is the velocity reached at that point, was discretized into “low” and “high” by means of the optimal cutoff identified from the ROC curve

	CTRCD = 0 (no) 243 (90)	CTRCD = 1 (yes) 27 (10)
*Age (years)*	57.43 ± 10.96	59.15 ± 8.86
*Age group*		
<50 50-64 65-79 ≥80	66 (27.16) 98 (40.33) 77 (31.69) 2 (0.82)	4 (14.81) 17 (62.96) 6 (22.22) 0 (0)
*LVEF (%)*	66.01 ± 7.24	62.42 ± 6.45
*LVEF group*		
<50% 50-54% >54%	6 (2.47) 6 (2.47) 231 (95.06)	1 (3.7) 0 (0) 26 (96.3)
*ACprev*		
0 (no) 1 (yes)	202 (90.18) 22 (9.82)	17 (70.83) 7 (29.17)
*antiHER2prev*		
0 (no) 1 (yes)	220 (98.21) 4 (1.79)	21 (87.5) 3 (12.5)
$\:{\varvec{t}}_{{\varvec{E}}^{\varvec{{\prime}}}}$	0.60 ± 0.08	0.63 ± 0.08
$\:{|\varvec{E}}^{\varvec{{\prime}}}|$	10.49 ± 2.95	9.17 ± 2.49
$\:{\varvec{v}}_{{\varvec{E}}^{\varvec{{\prime}}}}$	0.06 ± 0.03	0.07 ± 0.02
$\:{v}_{{E}^{{\prime\:}}}$ *group*		
Low High	95 (39.09) 148 (60.91)	1 (3.7) 26 (96.3)

### Comparison of TDI velocity functions

**Fig. 2:**
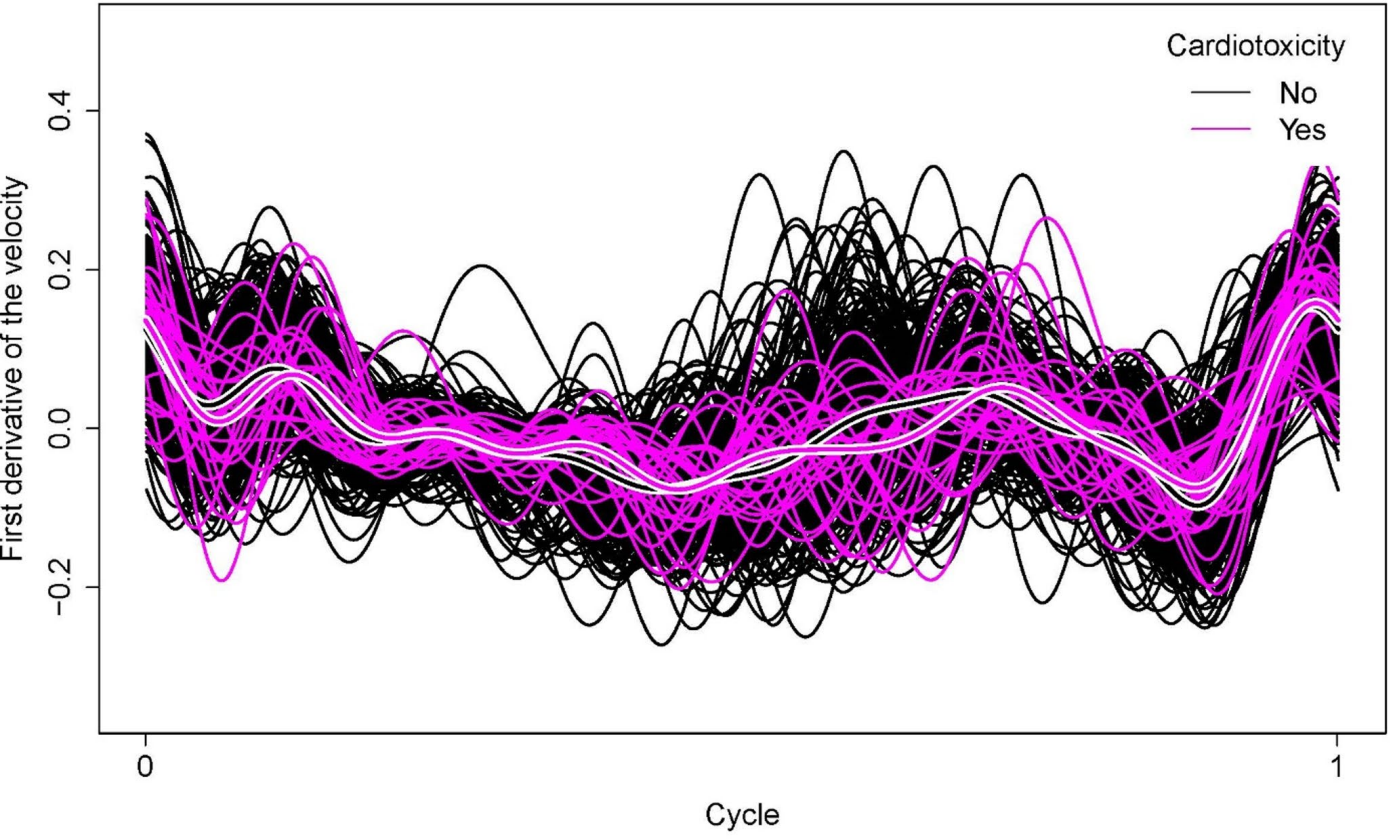
Derivated TDI cycles depending on the cardiotoxicity status. The derivative of the cycles of the patients who experienced cardiotoxicity during the follow-up period is shown in magenta. The derivative of the cycles of the patients who did not experience this side effect is shown in black. The thick lines with white borders represent the mean cycle of each group. The most notable differences between them are observed in the $\:{E}^{{\prime\:}}$ wave region

Since the cure indicator is only partially observed, estimating $\:{{\upmu\:}}_{0}\left(\text{t}\right)$ and $\:{{\upmu\:}}_{1}\left(\text{t}\right)$ from the data is challenging. In practice, the only available information is the CTRCD indicator (or censoring indicator), which indicates whether cardiotoxicity was observed during the follow-up period. Thus, $\:{{\upmu\:}}_{0}\left(\text{t}\right)$ can be estimated from the functions of the 27 patients who experienced cardiotoxicity during the follow-up period. In addition, to estimate $\:{{\upmu\:}}_{1}\left(\text{t}\right)$, 243 functions are available (the ones of the censored patients). The mean TDI function for patients without observed cardiotoxicity during the follow-up is shown as black with white borders in Fig. 1. In magenta with white borders, the mean function of patients who experienced cardiotoxicity is shown. These mean functions, which estimate $\:{{\upmu\:}}_{1}\left(\text{t}\right)$ and $\:{{\upmu\:}}_{0}\left(\text{t}\right)$, respectively, are quite similar around the S wave and at the end of the cycle. However, the differences are more remarkable around the $\:{E}^{{\prime\:}}$ wave. The same conclusion holds when considering the derivatives of the velocity functions (Fig. 2), which provides additional insight, as they highlight the rates of change in myocardial motion over the cardiac cycle.

### Statistical analysis of TDI functions

By applying the permutation test with $$\:{m}_{1}=27,$$$$\:{m}_{2}=243\:$$and $$\:B=10000$$, the obtained $$\:p$$-values were 0.032 ($$\:{D}_{1}$$), 0.028 ($$\:{D}_{2}$$) and 0.002 ($$\:{D}_{3}$$). For $$\:{D}_{1}$$ and $$\:{D}_{2}$$, the $$\:p$$-values decreased to 0.009 and 0.013, respectively, when focusing on the time interval [0.4, 0.9] of the cycle, that is, in a neighborhood of the $$\:{E}^{{\prime\:}}$$ wave. These results confirm that significant differences between the functions of both groups of patients were found around the $$\:{E}^{{\prime\:}}$$ wave, suggesting the potential predictive role of TDI in this context. In previous studies, a low $$\:{E}^{{\prime\:}}$$ wave peak was found to be associated with an increased risk of cardiotoxicity in individuals undergoing oncologic therapy [12–14]. However, to our knowledge, no prior evidence exists for the rightward displacement of the $$\:{E}^{{\prime\:}}$$ wave.

### Creation of a clinically applicable TDI parameter

Unfortunately, it is quite challenging to use TDI cycles in clinical practice because of the variability in ultrasound devices and the need for preprocessing. To simplify the interpretation of TDI data from the baseline echocardiogram, we propose focusing on two key points of the $\:{E}^{{\prime\:}}$ wave to create a predictive parameter:

- ${t}_{{E}^{\prime}}:$ the timing within the cycle (between 0 and 1) when the peak of the $\:{E}^{{\prime\:}}$ wave occurs.
- ${E}^{{\prime}}:$ the velocity reached at that point.

Quantitative analysis revealed that $\:{t}_{{E}^{{\prime\:}}}\:$was delayed in the CTRCD group (0.63 ± 0.08 vs. 0.60 ± 0.08), whereas the absolute $\:{E}^{{\prime\:}}$ velocity was lower in patients who developed CTRCD (9.17 ± 2.49 cm/s vs. 10.49 ± 2.95 cm/s). Taking this into account, we create a new variable related to $\:{E}^{{\prime\:}}$ (let us denote it by $\:{v}_{{E}^{{\prime\:}}}$), which is defined as follows: $\:{v}_{{E}^{{\prime\:}}}=\:\frac{{t}_{{E}^{{\prime\:}}}}{{|E}^{{\prime\:}}|}$. Women who will develop cardiotoxicity are expected to have a higher numerator (due to the rightward shift of the $\:{E}^{{\prime\:}}$ wave) and a lower denominator (since the peak is less pronounced). Therefore, the value of the new variable, $\:{v}_{{E}^{{\prime\:}}}$, tends to be higher for this population. That is, a high baseline value for this variable would be associated with cardiotoxicity. In the breast cancer dataset in [9], the mean value of $\:{v}_{{E}^{{\prime\:}}}$ for patients with cardiotoxicity was 0.07, whereas that for patients without cardiotoxicity was 0.06, with the value being significantly higher in the cardiotoxicity group ($\:p$-value = 0.002).

### ROC curve analysis and optimal cutoff value

A ROC curve was constructed to assess the ability of $\:{v}_{{E}^{{\prime\:}}}$ to discriminate between patients who developed cardiotoxicity and those who did not. The resulting obtaining an AUC was 0.6676. The optimal cutoff point for $\:{v}_{{E}^{{\prime\:}}}$, based on Youden’s index, was 0.052, with an associated sensitivity and specificity of 0.963 (96.3%) and 0.391 (39.1%), respectively.

### Cox regression analysis

To evaluate the effect of $\:{v}_{{E}^{{\prime\:}}}$ on cardiotoxicity, this variable was incorporated into a Cox regression model along with other variables commonly recognized as risk factors for cardiotoxicity. Three Cox models were fitted, and all were adjusted for discretized age and LVEF. Neither of these two variables was significant. The HRs and $\:p$-values associated with the other variables included in each model are shown in Table 2. The HRs for $\:{v}_{{E}^{{\prime\:}}}$ high (values > 0.052) represent the relative risk of cardiotoxicity compared with the reference group $\:{v}_{{E}^{{\prime\:}}}$ low (values ≤ 0.052). Similarly, the HRs for treatprev, ACprev, and antiHER2prev are compared to the reference category of no prior cardiotoxicity-related treatment. The results show that women with $\:{v}_{{E}^{{\prime\:}}}>0.052$ have a significantly higher risk of developing cardiotoxicity than those with lower values. Specifically, the HR for $\:{v}_{{E}^{{\prime\:}}}$ high in Model 1 was 13.35. Previous treatment also showed a significant association with cardiotoxicity in Model 1 (HR = 3.11, $\:p$-value = 0.00847). The C-index value for Model 1 was 0.756, indicating good discrimination between patients at risk of cardiotoxicity and those who are not. The details of Model 2 and Model 3 are shown in Table 2.

**Table 2:** Cox proportional hazard models were used to evaluate the effect of $\:{v}_{{E}^{{\prime\:}}}$ on cardiotoxicity. The three models were adjusted for age group and LVEF group. The variable $\:{v}_{{E}^{{\prime\:}}}=\:\frac{{t}_{{E}^{{\prime\:}}}}{{|E}^{{\prime\:}}|}$, where $\:{t}_{{E}^{{\prime\:}}}$ is the timing within the cycle (between 0 and 1) when the peak of the $\:{E}^{{\prime\:}}$ wave occurs, and $\:{|E}^{{\prime\:}}|$ is the velocity reached at that point, was discretized into “low” and “high” by means of the optimal cutoff identified from the ROC curve. The variables antiHER2prev, acprev and treatprev indicate that the patient received prior treatment with antiHER2 or anthracyclines or any of both, respectively

Model	Variable	HR (IC)	$\:\varvec{p}$-value
Model 1 (C-index 0.799)	$\:{v}_{{E}^{{\prime\:}}}$ high treatprev	13.35 (1.73, 103.26) 3.11 (1.34, 7.22)	0.01305 0.00847
Model 2 (C-index 0.796)	$\:{v}_{{E}^{{\prime\:}}}$ high ACprev antiHER2prev	14.91 (1.93, 115.27) 0.94 (1.02, 6.37) 0.76 (0.59, 7.82)	0.00961 0.04530 0.24355
Model 3 (C-index 0.756)	$\:{v}_{{E}^{{\prime\:}}}$ high	17.19 (2.27, 130.36)	0.00592

## Discussion

The key finding in this study is the proposal of a new echocardiographic parameter, $\:{v}_{{E}^{{\prime\:}}}$, which could serve as a predictor of a higher risk of moderate-to-severe CTRCD in patients with breast cancer who are scheduled to receive anti-HER2 therapies or anthracyclines. Specifically, values of $\:{v}_{{E}^{{\prime\:}}}\:$above the threshold of 0.052 can be used as a risk factor for CTRCD. This parameter is developed after identifying a rightward displacement of the $\:{E}^{{\prime\:}}$ wave in the baseline echocardiogram of patients who later developed cardiotoxicity during the follow-up period. While earlier studies have indicated that a low $\:{E}^{{\prime\:}}$ wave peak is linked to an elevated risk of cardiotoxicity [12–14], our research highlights the significance of timing this peak in the baseline evaluation, prior to initiating oncological treatment. The proposed quotient, $\:{v}_{{E}^{{\prime\:}}}$​, demonstrated a significant association with CTRCD, supporting the hypothesis that a slightly delayed $\:{E}^{{\prime\:}}$ wave, coupled with a low peak velocity, could serve as an early indicator of CTRCD in the baseline evaluation. These findings are also consistent with the graphical observations presented in Figs. 1 and 2, where notable differences between the two patient groups are evident around the $\:{E}^{{\prime\:}}$ wave.

In clinical practice, the initial risk assessment of patients who are going to receive anti-HER2 therapies or anthracyclines relies on clinical factors, cardiac biomarkers and echocardiography, with LVEF being the primary indicator [4]. The role of GLS in follow-up to detect early signs of subclinical dysfunction is well established [15]. However, the usefulness of this parameter in the baseline evaluation is less clear. Although lower baseline GLS values have been suggested as a potential risk factor for subsequent cardiotoxicity development, this relationship remains unclear, and currently no established GLS cutoff value has been identified as a risk factor in baseline echocardiograms [6].

In addition, recent research has shown that a decrease in several left atrial strain parameters could be a predictor of cardiotoxicity in patients with HER2-positive breast cancer receiving trastuzumab [16]. Additional studies have further explored atrial strain assessment methods for early detection of cardiac dysfunction [17, 18]. However, the focus of these findings is on changes during follow-up rather than the use of baseline values as predictors. Furthermore, left atrial strain analysis, similar to GLS, requires specialized software that is not routinely available in all hospitals, whereas $\:{E}^{{\prime\:}}$ wave acquisition is part of the standard echocardiographic evaluation.

Improving the baseline risk stratification of CTRCD would allow for more personalized monitoring, with a focus on patients with higher risk, and would allow for the optimization of resources.

### Study limitations

As previously mentioned, the use of TDI cycles in clinical practice remains challenging. To obtain such data, TDI images need to be preprocessed, and the algorithm proposed in the literature [10] is tailored to a specific type of TDI image. The configuration and appearance of TDI images depend on the ultrasound device used. Not all hospitals use the same brands, and it is common for ultrasound machines to be replaced over time. Therefore, owing to this heterogeneity, it is very difficult to consistently rely on a suitable preprocessing algorithm. To address this challenge in practice, we propose focusing on key points of the $\:{E}^{{\prime\:}}$ wave.

Another limitation of our study is the absence of GLS data for comparison. However, it is important to emphasize that our approach with TDI parameters is intended to be complementary rather than alternative to GLS. The $\:{v}_{{E}^{{\prime\:}}}$​ parameter we propose addresses a specific clinical need: identifying high-risk patients during baseline evaluation before treatment initiation, whereas GLS is particularly valuable for detecting subclinical cardiac dysfunction during follow-up once treatment has started. Our research does not suggest replacing GLS monitoring during follow-up, which remains essential for early detection of developing cardiotoxicity [6, 19, 20]. Instead, we propose that TDI assessment at baseline could enhance initial risk stratification. An optimal approach would incorporate both: baseline risk assessment using conventional parameters and TDI-derived measures such as $\:{v}_{{E}^{{\prime\:}}}$​, followed by GLS monitoring during treatment to detect subclinical changes in cardiac function.

Although our findings highlight the potential role of high $\:{v}_{{E}^{{\prime\:}}}$ as a promising echocardiographic predictor for cardiotoxicity, the wide confidence intervals of the hazard ratios observed in the Cox models suggest considerable uncertainty with respect to the precision of the estimates. This may be due to the small number of cardiotoxicity events in our dataset (27 out of 270 patients), a limitation inherent to most CTRCD studies. This proportion is consistent with the typical incidence of CTRCD reported in the literature. Future studies with larger sample sizes and external validation are necessary to strengthen our findings.

In addition, only patients with sinus rhythm and regular RR were considered in this study. Therefore, the results might not be applicable to patients with atrial fibrillation at baseline.

## Conclusions

Clinical images, such as those obtained by TDI, can contain valuable predictive information for patients with breast cancer who are receiving anthracyclines of antiHER2 therapies. With further research, $\:{v}_{{E}^{{\prime\:}}}$, which is the ratio between the timing of the $\:{E}^{{\prime\:}}$ wave peak within the cycle and the corresponding peak velocity (in absolute value), could be a useful variable for predicting the risk of cardiotoxicity. The significant advantage of this approach is that the analysis is performed on baseline echocardiograms prior to oncological treatment, potentially allowing for early risk stratification and more personalized monitoring strategies for patients at greater risk of developing CTRCD.

## Data Availability

No datasets were generated or analysed during the current study.
